# Visualisation of chicken macrophages using transgenic reporter genes: insights into the development of the avian macrophage lineage

**DOI:** 10.1242/dev.105593

**Published:** 2014-08

**Authors:** Adam Balic, Carla Garcia-Morales, Lonneke Vervelde, Hazel Gilhooley, Adrian Sherman, Valerie Garceau, Maria W. Gutowska, David W. Burt, Pete Kaiser, David A. Hume, Helen M. Sang

**Affiliations:** The Roslin Institute and Royal (Dick) School of Veterinary Sciences, University of Edinburgh, Easter Bush, Midlothian EH25 9RG, UK

**Keywords:** Chicken, Dendritic cells, Embryonic development, Immunity, Macrophages, Transgenics

## Abstract

We have generated the first transgenic chickens in which reporter genes are expressed in a specific immune cell lineage, based upon control elements of the colony stimulating factor 1 receptor (*CSF1R*) locus. The Fms intronic regulatory element (FIRE) within *CSF1R* is shown to be highly conserved in amniotes and absolutely required for myeloid-restricted expression of fluorescent reporter genes. As in mammals, *CSF1R*-reporter genes were specifically expressed at high levels in cells of the macrophage lineage and at a much lower level in granulocytes. The cell lineage specificity of reporter gene expression was confirmed by demonstration of coincident expression with the endogenous CSF1R protein. In transgenic birds, expression of the reporter gene provided a defined marker for macrophage-lineage cells, identifying the earliest stages in the yolk sac, throughout embryonic development and in all adult tissues. The reporter genes permit detailed and dynamic visualisation of embryonic chicken macrophages. Chicken embryonic macrophages are not recruited to incisional wounds, but are able to recognise and phagocytose microbial antigens.

## INTRODUCTION

Macrophages participate in a wide range of processes during embryonic development and throughout life, including organogenesis and homeostasis, clearance of apoptotic cells, pathogen recognition, phagocytosis and destructions of pathogens, and antigen presentation (Pollard, 2009; Jones and Ricardo, 2013; Wynn et al., 2013). Chicken and quail embryos are widely used as models of amniote development because of the ease with which embryos can be manipulated and visualised (Stern, 2005; Sauka-Spengler and Barembaum, 2008; Le Douarin et al., 1994). Avian embryonic macrophages have been shown to have diverse roles, including phagocytosis of dead cells (Cuadros et al., 1992), remodelling of the eye primordium (Martín-Partido and Navascués, 1990; Martín-Partido et al., 1991), guidance of axonal growth and vascular development in the central nervous system (Cuadros et al., 1993), and the development of lymphoid tissues (Houssaint, 1987).

The mononuclear phagocyte system in mammals is a family of cells derived from a shared progenitor, and includes blood monocytes, tissue macrophages and classical dendritic cells. These cells are found throughout the body and can be detected by immunocytochemical localisation of lineage-restricted surface markers (Hume, 2006). Delineation of the chicken mononuclear phagocyte system in embryonic development and in adult birds has been hampered by the lack of available reagents for specific molecular targets and by significant differences in their biology. Chickens lack lymph nodes (McCorkle et al., 1979) and lymphoid tissues with equivalent function are difficult to visualise and isolate, which makes the isolation of cells and analysis of local immune responses challenging.

The differentiation, proliferation and survival of macrophages in mammals is controlled primarily by the cytokine macrophage colony stimulating factor (MCSF or CSF1) through its interaction with CSF1R, the product of the *c-FMS* proto-oncogene (Chitu and Stanley, 2006; Hume and MacDonald, 2012). A second ligand of CSF1R, interleukin 34 (IL34), has a more spatially restricted expression profile in embryos and contributes to the maintenance of specific macrophage subpopulations (Nakamichi et al., 2013). CSF1, CSF1R and IL34 are functionally conserved in birds (Garceau et al., 2010). Recently, we produced a monoclonal antibody to chicken CSF1R that labels monocytes and tissue macrophages (Garcia-Morales et al., 2013). CSF1R gene orthologues have been identified in all vertebrates studied to date, although their function may not be absolutely conserved. In fish there is a duplication of CSF1 and CSF1R loci and the receptor is expressed in both neural crest-derived xanthophores and macrophages (Wang et al., 2013).

The murine *Csf1r* genomic sequence contains a conserved regulatory element, the Fms-intronic regulatory element (FIRE), that is essential for macrophage-specific expression of reporter genes *in vitro* and *in vivo* (Himes et al., 2001; Sasmono et al., 2003). A segment of genomic DNA containing both the Csf1r promoter and FIRE sequence is sufficient to drive expression of green fluorescent protein (eGFP) specifically in all macrophage lineage cells in transgenic mice (Sasmono et al., 2003; Ovchinnikov et al., 2010). These ‘MacGreen’ mice have been used extensively in functional genomics and fate-mapping in mice (Burke et al., 2008; Ebert et al., 2009; MacDonald et al., 2010; Mooney et al., 2010; Lilja et al., 2013).

In this study, we show that FIRE is present in all amniote lineages examined to date and describe the generation of transgenic chicken reporter gene lines in which the chicken *CSF1R* promoter and FIRE enhancer sequences are linked to green or red fluorescent reporter proteins. The lineage-restricted expression of these reporter genes confirms the conserved function of FIRE from birds to mammals. We show that embryos from the macrophage reporter lines can be used to visualise the dynamic behaviour of macrophages in the developing embryo. Chicken embryonic macrophages accumulate in regions of cell death but do not respond to wounding, are able to recognise and phagocytose microbial antigens, and to undergo local proliferation in tissues. In post-hatch birds we use the *CSF1R*-reporter gene to define the phenotype of blood monocytes and examine the diversity of the mononuclear phagocyte system in lymphoid and other tissues. Finally, we show that the brightness and specificity of the *CSF1R*-reporter gene expression gives a unique macroscopic view of the organisation and extent of chicken lymphoid tissues.

## RESULTS

### The first intron of the avian *CSF1R* gene contains a conserved enhancer element

**Fig. 1.**
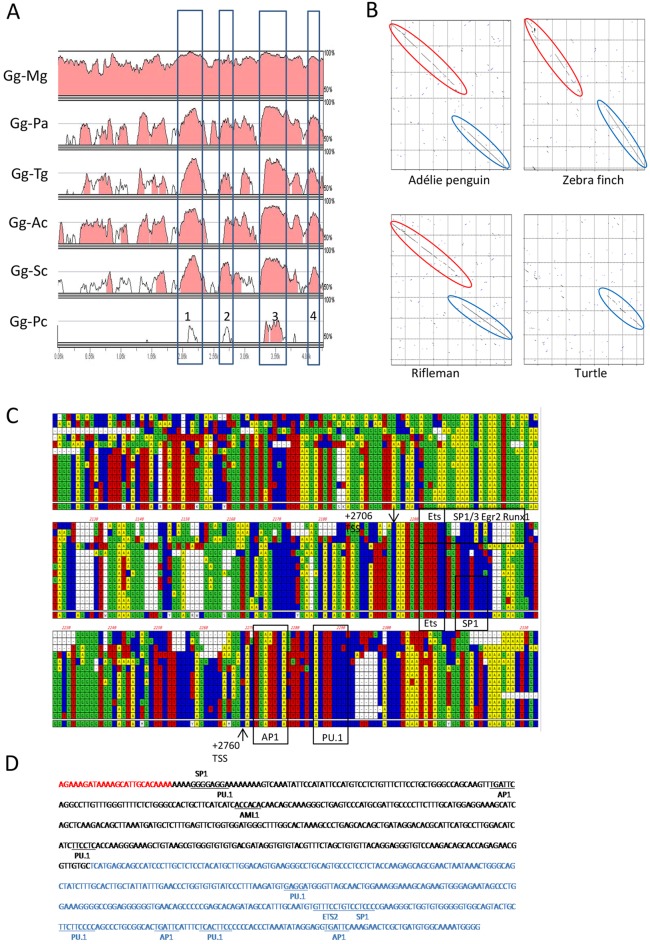
**Identification of putative macrophage lineage-specific regulatory elements in the first intron of the chicken *CSF1R* gene.** (A) mVista alignment (http://gsd.lbl.gov/vista/) of the *CSF1R* first intron comparing chicken (Gg) with turkey (Mg), Adélie penguin (Pa), zebrafinch (Tg), rifleman (Ac), ostrich (Sc) and Chinese softshell turtle (Pc). Conserved regions (>70% homology over 100 bp window) are shaded. The positions of four major conserved non-coding elements (CNEs) are boxed and numbered. (B) Pustell DNA matrix alignment of the avian/reptile *CSF1R* CNE2 and CNE3. The unbroken diagonal lines represent regions of high sequence conservation, and the broken and offset lines indicate that an insertion has occurred in the chicken/turkey lineage in comparison with the other species shown here. The avian-specific CNE2 is highlighted in red; the CNE-3, which is conserved in birds and turtle sequence, is highlighted in blue. (C) Alignment of mammalian Fms-intronic regulatory element (FIRE) with the *CSF1R* CNE3 region in birds/reptiles. Species sequences from top to bottom are human, mouse, platypus, turtle, alligator, Adélie penguin, budgerigar, ostrich, rifleman, zebrafinch, duck, turkey, chicken and consensus sequence. Arrows indicate the location of the two murine FIRE transcription start sites (Sauter et al., 2013) and conserved transcription factor binding sites are also shown. (D) Sequence of the chicken macrophage lineage-specific regulatory element used in this study: binding sites for PU.1, C/EBP, AP1, SP1 and AML1 are identiﬁed. The avian-specific CNE2 is highlighted in red and the avian-reptile-mammal conserved CNE3 is in blue.

Conservation of sequences within the first intron of avian *CSF1R* genes was evident from an alignment of chicken and zebrafinch *CSF1R* genomic sequences (Garceau et al., 2010). The availability of many more genome sequences has enabled us to align sequences of four additional bird species and a reptile with chicken, to identify potential regulatory sequences in the chicken by their conservation between distantly related species. The first intron of *CSFIR* contains four conserved non-coding elements (CNEs) that are present in all birds (Fig. 1A). Pustell DNA matrix alignment of CNE2 and CNE3 suggests that they were formed in the galliforme lineage by an insertion into an original single CNE (Fig. 1B). CNE3 is also conserved in turtles (Fig. 1A,B). Comparison of mammalian FIRE with CNE3 in birds and reptiles identified several regions of ultra-conserved sequence (Fig. 1C). These ultra-conserved regions contain the precise binding sites of transcription factors AP1 and PU.1 that are occupied in the macrophage nucleus (Tagoh et al., 2002) and are required for macrophage lineage-specific transcription of *Csf1r* in mice (Fig. 1C,D) (Sauter et al., 2013). To test the function of the candidate chicken FIRE sequence, we produced eGFP reporter constructs containing the chicken *CSF1R* promoter region (Garceau et al., 2010) with or without the CNE3 region (supplementary material Fig. S1A,B). eGFP expression was detected in stably transfected HD11 macrophage cells only when CNE3 was included, whereas no expression was detected in transfected DF-1 fibroblast cells (supplementary material Fig. S1C). Based upon sequence conservation and function, we refer to CNE-3 as chicken FIRE.

### FIRE is required for macrophage-restricted expression in transgenic birds

We developed HIV vectors carrying the chicken *CSF1R* regulatory sequences directing expression of eGFP or the red fluorescent protein mApple to the cytoplasm of macrophages and used these to generate transgenic chickens (McGrew et al., 2004). The transgenes contain splice donor and acceptor sites flanking FIRE, to reproduce the structure of the native *CSF1R* gene (supplementary material Fig. S1D). Fortuitously, this approach resulted in deletion of FIRE in the majority of transgenic birds hatched, as a result of splicing events during the production of lentiviral vector genomic RNA (supplementary material Fig. S2A,B). There was no evidence of reporter gene expression in any of the individual transgenic birds in which FIRE was deleted (supplementary material Fig. S2C), confirming the essential role of the FIRE sequence in expression. We established transgenic lines from birds carrying the intact transgenes, named MacRed (mAPPLE-expressing) and MacGreen (eGFP-expressing), collectively MacReporter chickens, and used these to examine lineage specificity of the transgene expression.

### *CSF1R*-transgene expression identifies macrophages in chicken embryos

**Fig. 2.**
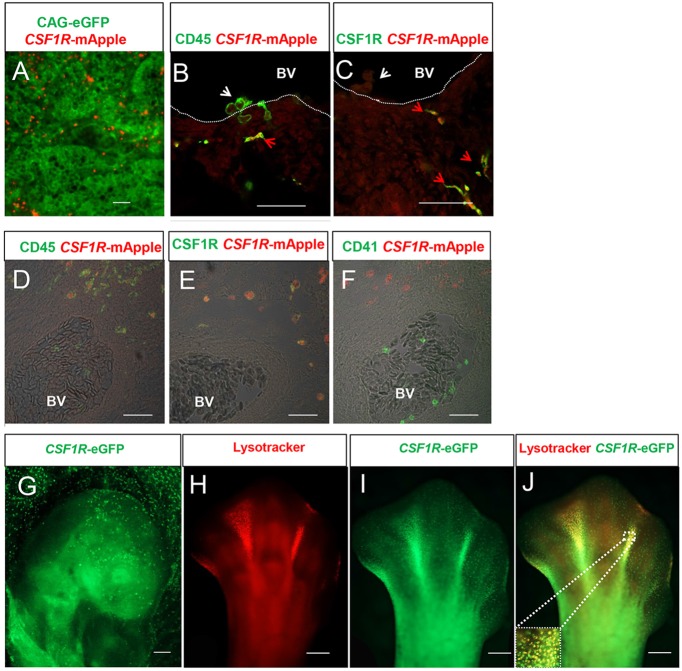
***CSF1R*-transgene expression is restricted to macrophages in MacReporter embryos.** (A) *CSF1R*-mApple^+^ cells (red) are restricted to the lumen of primitive blood vessels in ubiquitous *CAG*-eGFP-expressing HH13 stage embryos (green). (B,C) Confocal analysis of transgene expression in HH21 stage *CSF1R*-mApple embryos indicates that transgene expression is restricted to CD45^+^ (B, green), CSF1R^+^ (C, green) cells in the mesenchyme (red arrowheads) and not CD45^+^ cells budding from the epithelial layer of the dorsal aorta (white arrowheads). Dotted lines mark the blood vessel (BV) lumen. Scale bars in A-C: 100 µm. (D-F) Confocal analysis of CSF1R staining (green) of *CSF1R*-mApple transgene-expressing cells (red) in the mesenchyme tissue of a HH29 embryo. The transgene is expressed in cells (red) that are CD45^+^ (D, green) and CSF1R^+^ (E, green), but are CD41/61^−^ (F, green). Scale bars in D-F: 100 µm. BV, blood vessel lumen. (G) Scattered eGFP^+^ cells are found in the embryonic (Emb.) and extra-exbryonic (Ex-Emb) tissues of HH15 MacGreen embryos. Scale bar: 200 µm. (H-J) Colocalization of eGFP^+^ cells with LysoTracker Red-stained lysosomes in HH33 embryo footplate and in the interdigit region. Inset in J shows the boxed area in more detail. Scale bars in G-J: 200 µm.

The distribution and phenotype of *CSF1R*-transgene expressing cells was examined in chicken embryos from the MacRed and MacGreen transgenic lines. Yolk sac-derived macrophages and erythrocytes are the earliest haematopoietic cell lineages to develop in the chick. Recognisable blood islands containing Runx1^+^ haematopoietic progenitors have been detected in HH5 stage embryos (Bollerot et al., 2005), but the first *CSF1R*-transgene-expressing cells appeared in the yolk sac at HH13. These cells were confined to the lumen of primitive blood vessels (Fig. 2A). The pattern of emergence is consistent with previous reports of the earliest appearance of macrophages in the chicken embryo (Cuadros et al., 1992). Neither CSF1R protein nor transgene expression was detected in erythrocytes or definitive haematopoietic stem cell clusters budding from the floor of the dorsal aorta in HH21 stage embryos. *CSF1R*-transgene expression was confined to a ramified CSF1R^+^ cell population that co-expressed the haematopoietic cell marker CD45 (Fig. 2B,C). Hence, the *CSF1R*-transgene expression was restricted to macrophages in the early chicken embryo prior to the emergence of other myeloid cell lineages. Thrombocytes, which are nucleated in birds, appear first in HH29 stage embryos. Thrombocytes also lacked any detectable expression the reporter transgene (Fig. 2D-F).

*CSF1R-*transgene expressing cells were widely distributed in developing embryos in a speckled pattern (Fig. 2G), consistent with the distribution of CSF1R mRNA in chicken embryos (Garceau et al., 2010) and earlier studies of phagocytic cells in the chicken embryo (Cuadros et al., 1992). The cells were visible throughout the body and concentrated as expected in areas of programmed cell death (Rotello et al., 1994; Hopkinson-Woolley et al., 1994), such as the interdigit regions of stage HH33 embryo leg buds (Fig. 2H-J). Embryos from the MacRed and MacGreen lines showed identical distributions of fluorescent cells (not shown). LysoTrackerRed (LyTRd), a dye that accumulates in phagolysosomes, co-stained eGFP-expressing cells in areas of programmed cell death in the leg buds, confirming the likely phagocytic function of *CSF1R*-transgene-expressing cells (Fig. 2J). Nevertheless, eGFP-expressing cells outside the regions of programmed cell death did not stain with LyTRd, suggesting that labelling of lysosomal compartments underestimates embryonic macrophage numbers.

### Visualisation of the response of embryonic chicken macrophages to wounding

**Fig. 3.**
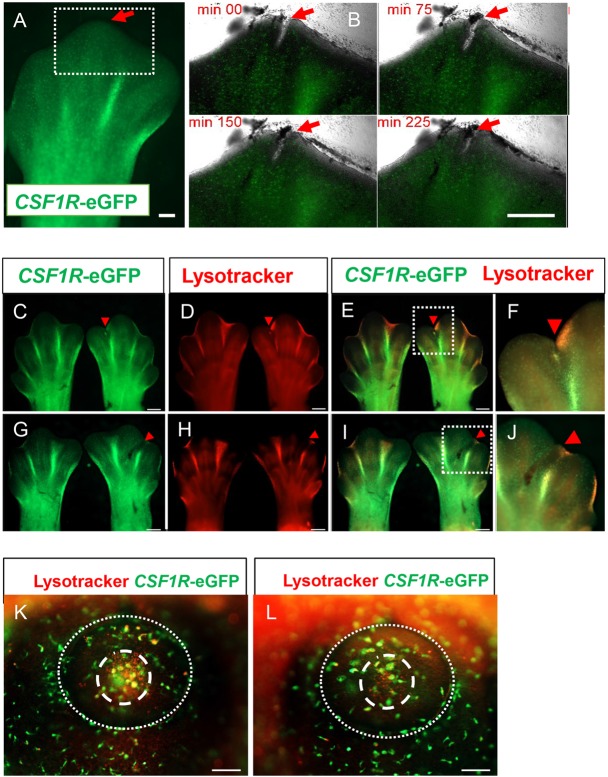
**Embryonic macrophages are not recruited to wounds.** (A,B) Time-lapse imaging of embryonic macrophage response to incisional wounding in the footpad of HH31 stage embryos *in vitro*. The tip of the central digit of a footpad (A, red arrow) was wounded with an ultrafine tungsten needle. Scale bar: 200 µm. Subsequent panels (B) focus on the behaviour of macrophages in the region of the incisional wound (boxed area). (B) No recruitment of macrophages to the wound (red arrows) is observed. Scale bar: 500 µm. (C-L) *In ovo* macrophage response to wounding. LysoTrackerRed (LyTRd) staining of *CSF1R*-eGFP embryonic limb buds 24 h after incisional (C-F) or crush (G-J) wounding of HH31 embryonic limb buds. Wounded limb buds are on the right of each panel and control contralateral limb buds are shown on the left. Red arrowheads indicate site of wounding and boxed areas (E,I) show details of the wound site in F,J. Compared with the contralateral control limb bud, there is no accumulation of macrophages at the wound site (red arrowheads), and diminishment of macrophage accumulation in the interdigit region adjacent to the wound is apparent (E,F,I,J). Scale bars: 500 µm. (K,L) LyTRd staining of eye primordium of *CSF1R*-eGFP embryos wounded in the eye primodium at HH16 *in ovo*. There is no obvious recruitment of macrophages with lysosomes in the wounded (L) compared with unwounded (K) eye primordium, although LyTRd staining indicates a region of cell death (dashed circle) in the centre of the lens vesicle (dotted line). Scale bars: 100 µm.

In embryonic zebrafish and *Xenopus*, macrophages are rapidly recruited to wound sites (Mathias et al., 2009; Costa et al., 2008), whereas this does not occur in mouse embryos until late in development (Hopkinson-Woolley et al., 1994). We used the transgenic lines to investigate the response to wounding using an organ culture of limb buds and after limb bud wounding *in ovo*. In organ-cultured limb buds, the wound gradually closed over a 4 h period following an incision (Fig. 3A,B). Although macrophages in the limb bud were highly motile and observed in the immediate vicinity of the wound, no recruitment to the wound site was seen (Fig. 3A,B). No accumulation of macrophages at the wound site was observed 24 h after wounding *in ovo* (Fig. 3C-J) and in some instances a reduction in interdigit macrophages was observed after incisional wounding (Fig. 3C-F). Similarly, in an eye wound model (supplementary material Movie 1), macrophages were observed in the immediate area of the wound (supplementary material Movie 1, red arrow), but there was no recruitment of macrophages to the wound site during the period of imaging. No accumulation occurred even after 24 h, despite evidence of phagocytosis of apoptotic cells in foci of programmed cell death such as the centre of the lens vesicle (Fig. 3K,L).

### Visualisation of the response of macrophages to microbial antigen in the embryonic vasculature

**Fig. 4.**
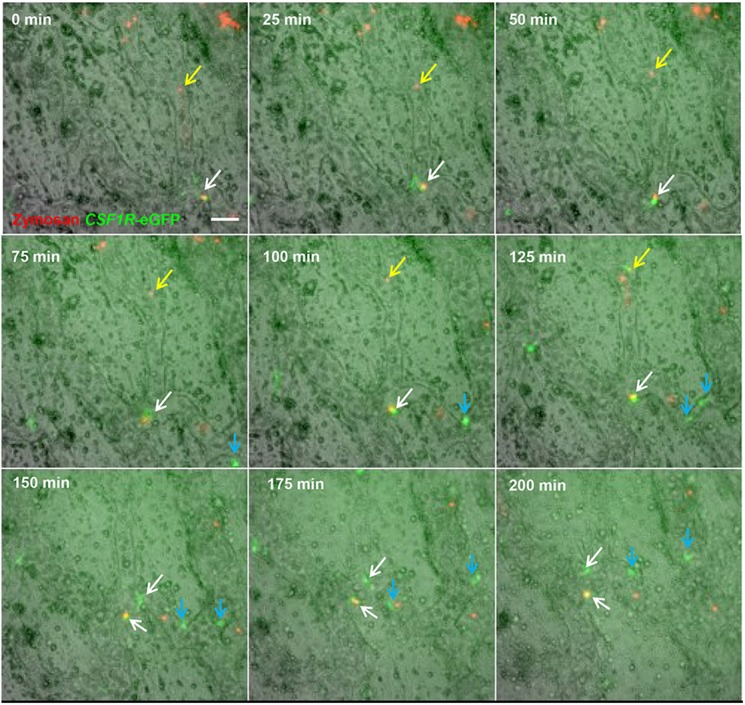
**Macrophages associated with the embryonic vasculature are highly motile and phagocytic, and undergo local division.** Time-lapse imaging of region above the vitelline artery near the embryo proper. The aorta of *CSF1R*-eGFP embryos was injected with Texas Red-labelled zymosan 1 h prior to the beginning of imaging. Most zymosan particles adhered to the blood vessel walls (yellow arrows). eGFP^+^ macrophages are highly motile. Between 100 and 125 min from the start of filming, a zymosan particle (yellow arrow) becomes associated with a macrophage; this macrophage re-enters the circulation, removing the zymosan particle by 150 min. At 0 min, a zymosan particle is contained within a macrophage (white arrow); from 0-75 min this macrophage is both motile and exhibits changes in morphology. At 100 min, this macrophage (white arrow) no longer exhibits movement and does not extend any cellular processes. A similar macrophage without a phagocytised zymosan particle (blue arrow) exhibits identical behaviour. At 100-150 min, both undergo division (white and blue arrows), and daughter cells resume active patrolling of the vasculature. Scale bar: 50 µm.

Vitelline vasculature macrophages imaged in HH17 MacGreen embryos were highly motile and were observed both within blood vessels and in a perivascular locations but not integrated into the blood vessel walls, as described by Al-Roubaie et al. (2012). Within blood vessels, macrophages were observed crawling on the blood vessel walls, both as isolated cells and as cell clusters (supplementary material Movie 2). This crawling behaviour is reminiscent of ‘patrolling’ behaviour reported for a subset of blood monocytes in mice that respond to microbial infection (Auffray et al., 2007). A well-established model for studying the interactions of microbes with phagocytes is the recognition and phagocytosis of microbial-derived zymosan particles (Underhill, 2003). We determined the capacity of patrolling macrophages within the vitelline blood vessels to recognise and phagocytose zymosan particles by injection of Texas Red-labelled zymosan particles into the dorsal aorta of HH17 MacGreen embryos. These particles were observed throughout the embryonic and extra-embryonic vasculature where they stuck to the blood vessel walls. Patrolling macrophages moved towards and engulfed zymosan particles, and then either continued to crawl along the vessel walls or entered the circulation (Fig. 4; supplementary material Movie 3). Cell division of patrolling embryonic macrophages associated with the vasculature was frequently observed and macrophages containing zymosan particles were also divided. This process involved the cessation of patrolling behaviour, retraction of cellular processes and rounding of cells before cell division. After cell division, both daughter cells resumed a ramified morphology and patrolling behaviour (Fig. 4; supplementary material Movie 3), indicating that mature yolk sac-derived macrophages are a self-renewing population.

### *CSF1R*-transgene expression identifies macrophages and other cells of the mononuclear phagocyte system in post-hatch chickens

There is no unequivocal marker for the chicken mononuclear phagocyte system, and the relationship between many key members of this family of cells remains unclear (Igyártó et al., 2007; Del Cacho et al., 2008). In mammals, monocytes, the circulating members of the mononuclear phagocyte system, can be divided into several subsets (Wong et al., 2012). In chickens, only a single subset has been reported (Mast et al., 1998). In FACS analysis of chicken blood, cells that expressed high levels of mApple co-expressed the known monocyte-restricted marker KUL01 (Mast et al., 1998) and CSF1R (supplementary material Fig. S3A). No transgene expression was detected in T-cells (CD3^+^) or B-cells (Bu-1^+^) (supplementary material Fig. S3A). The transgene-expressing chicken monocytes exhibited relatively uniform surface labelling with anti-CD45, MHCII and CD11, and somewhat greater diversity of αIIb β3 integrin, CD41/61. In MacGreen mice, the *Csf1r*-eGFP reporter can be detected in inflammatory neutrophils, which express Csf1r mRNA, but not the protein product (Sasmono et al., 2007). Birds do not have neutrophils, the equivalent cell population being heterophils (Caxton-Martins and Daimon, 1976). Transgene expression and cell-surface CSF1R were both detectable in this cell subset (supplementary material Fig. S3B), but at a level approximately one-tenth of that in the monocytes.

**Fig. 5.**
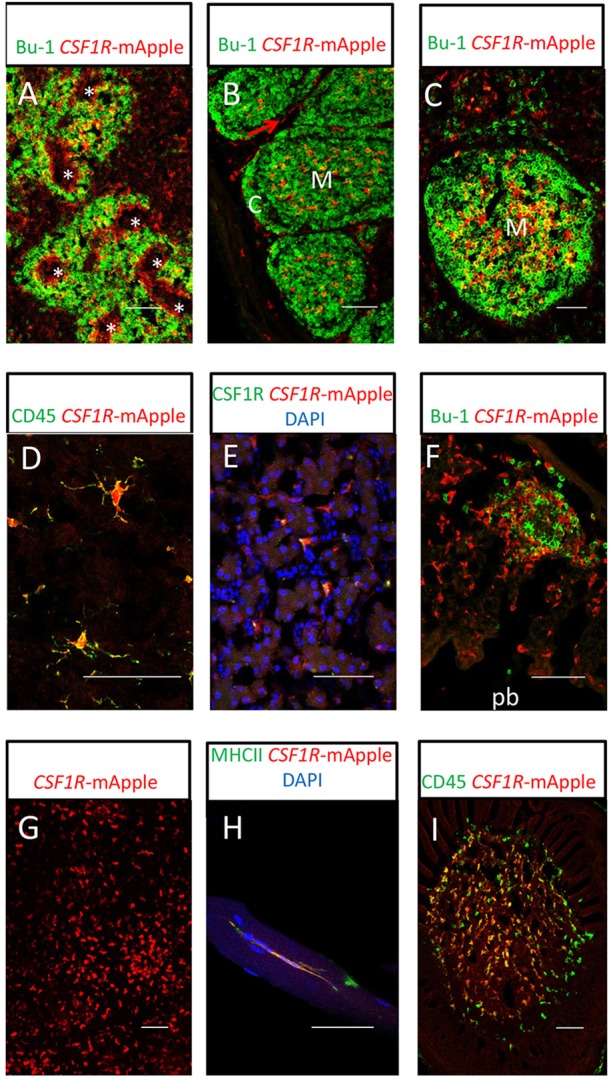
**Confocal analysis of MacRed chicken post-hatch mononuclear phagocyte populations.** (A) Splenic mononuclear phagocytes (red) and Bu-1^+^ B-cells (green) from a 16-week-old MacRed chicken. Rings of transgene-expressing cells can clearly be seen surrounding the ellipsoid (asterisk). (B) Bursa of Fabricius from an 8-day-old MacRed chicken: Bu-1^+^ B cells (green) show arrangement of the B-cell follicles; mononuclear phagocytes (red) are present in the medulla (M) and interfollicular region (red arrow), but not in the cortex (C) of B-cell follicles in the bursa of Fabricius. (C) Caecal tonsil B-cell follicle from a 10-week-old MacRed chicken, showing location of mononuclear phagocytes (red) and Bu-1^+^ B-cells (green). Transgene-expressing cells concentrated in the medulla region (M) of the B-cell follicle are a dense network of FDC. (D) Microglial cells (red) in the cerebellum of an 8-day-old MacRed chicken showing colocalisation with CD45 staining (green). (E) Kupffer cells (red) showing colocalisation with CSF1R (green) from a 13-week-old MacRed chicken liver. (F) Lung mononuclear phagocytes (red) and Bu-1^+^ B-cells (green) in the interstitial tissue of the parabronchial wall from a 16-week-old MacRed chicken. The parabronchial lumen (pb) is indicated. (G) Epidermal mononuclear phagocyte cells (red) in epidermal sheet preparation from a 10-week-old MacRed chicken. (H) Breast muscle mononuclear phagocytes (red) from a 16-week-old MacRed chicken co-expressing MHCII (green). (I) Feather pulp mononuclear phagocytes from an 8-day-old MacRed chicken (red) co-stained with CD45 (green). Scale bars: 50 µm.

The expression of *CSF1R*-transgene expression in chicken tissue mononuclear subsets in the lymphoid organs and non-lymphoid tissues was examined by confocal microscopy. In the spleen, *CSF1R*-transgene-expressing cells were abundant and found in association with B-cells of the peri-ellipsoid lymphocyte sheath (PELS) and within the ellipsoid (Fig. 5A), consistent with previous studies of splenic macrophage populations (Jeurissen et al., 1989; Nagy et al., 2005; Igyártó et al., 2007). In the bursa of Fabricius, the avian-specific primary lymphoid organ for B-cell production, *CSF1R*-transgene-expressing cells were found in the medulla of B-cell follicles and in the interfollicular tissues (Fig. 5B). The location of *CSF1R*-transgene-expressing cells in the medulla is consistent with their identity as bursal secretory dendritic cells (BSDCs) (Oláh et al., 1992). Dense networks of *CSF1R*-transgene-expressing cells were present in the medulla region of germinal centres in the caecal tonsil (Fig. 5C). The distribution of cells in the medulla of germinal centres is consistent with cells previously described as avian follicular dendritic cells (FDCs) (Eikelenboom et al., 1983; Jeurissen, 1993). Both BSDCs and FDCs expressed high levels of CSF1R protein (supplementary material Fig. S4).

**Fig. 6.**
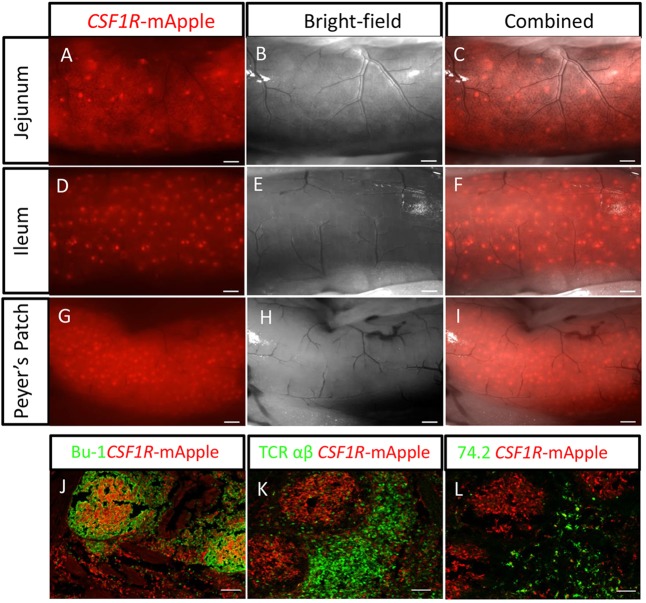
**F distribution of lymphoid aggregates in the MacRed chicken gut.** (A-I) External views of different regions of a 1-year-old MacRed chicken showing several scattered lymphoid aggregates in the jejunum (A-C), numerous scattered lymphoid aggregates in the ileum (D-F) and a high concentration of lymphoid aggregates in the ileum Peyer's Patch. Scale bars in A-I: 500 µm. (J-L) Immunofluorescence staining of Peyer's patches showing organisation of *CSF1R*-mApple-expressing cells (red) in relation to: (J) Bu-1^+^ B-cells (green), (K) TCR αβ (Vβ1)^+^ T-cells (green) and (L) CVI-ChNL-74.2^+^ macrophages (green). Scale bars in J-L: 100 µm.

We observed *CSF1R*-transgene-expressing cells in the brain (Fig. 5D). Their CD45^+^ phenotype and highly ramified appearance is consistent with their identity as microglial cells (Cuadros et al., 2006), the resident macrophage population of neuronal tissues. Similarly, macrophages of the liver (Kupffer cells) were located in the sinusoids, as expected (Fig. 5E). In contrast to mammalian lung, the avian lung does not contain alveoli or cells equivalent to alveolar macrophages, but there is a network of phagocytes surrounding the larger airways (de Geus et al., 2012). Consistent with this pattern, *CSF1R*-transgene-expressing cells were scattered throughout the interstitial tissue of the parabronchial wall and clustered with B-cells to form small, isolated lymphoid follicles in the lung (Fig. 5F). Epidermal sheet preparations contained large numbers of transgene-expressing cells, both scattered cells and in small clusters (Fig. 5G), consistent with reported distribution of chicken Langerhans cells (Igyártó et al., 2006). Unexpectedly, in the skeletal muscle we observed many *CSF1R-*transgene-expressing cells. These cells co-expressed class II MHC (Fig. 6H) and were also positive for CSF1R (not shown), indicating they are resident skeletal muscle macrophages. One other macrophage population that is unique to birds is in the skin, where the transgene highlighted the major haematopoietic cell subset in feather pulp (Fig. 5I).

### Identification of widely distributed lymphoid structures highlighted by *CSF1R*-transgene expression

In post-hatch chicken, the bulk of lymphoid tissue consists of solitary or aggregated lymphoid follicles, which are difficult to identify (Vaughn et al., 2006). This severely limits the study of lymphoid tissue development and local immune responses in avian compared with mammalian models. The post-hatch development of these lymphoid follicles varies with time and between individual chickens (Befus et al., 1980). The lymphoid follicles were readily identified in the gut tissues of MacReporter chicken, as aggregates of *CSF1R*-transgene-expressing cells ranging from single isolated aggregates to structures composed of hundreds of aggregates (Fig. 6A-I). The aggregates of *CSF1R*-transgene-expressing cells were found within organised lymphoid structures, typically comprising a B-cell-dominated germinal centre surrounded by a T-cell-rich area of tissue. Transgene-expressing cells formed dense networks of cells within the medulla region of the B-cell zone of the germinal centres (Fig. 6J-L). The distribution of *CSF1R*-transgene-expressing cells in the medulla of germinal centres is consistent with cells previously described as avian FDCs (Eikelenboom et al., 1983; Jeurissen, 1993). Scattered cells were also detected throughout T-cell zones (Fig. 5K). The reporter colocalised with the antigen bound by antibody CVI-ChNL-74.2, which recognises both red pulp macrophages and a ring of macrophages surrounding the peri-ellipsoid lymphocyte sheath (Jeurissen et al., 1992). Cells co-expressing the reporter and this marker were excluded from the B-cell follicles, but were concentrated in T-cell-rich regions (Fig. 6L).

## DISCUSSION

In mice, restriction of *Csf1r* expression to macrophages is dependent on the intronic enhancer element FIRE (Sasmono et al., 2003). The present study demonstrates that FIRE is conserved across species at both the sequence level and in its function in macrophage expression. *CSF1R* FIRE probably appeared in an early amniote, before the separation of the synapsids (mammals) and sauropsids (birds and reptiles), between 320 and 340 million years ago. We have shown elsewhere that mouse FIRE is active as a macrophage-specific enhancer in a wide range of mammals and birds (Pridans et al., 2014).

We have demonstrated the specificity of *CSF1R*-transgene expression in the MacReporter lines, and their utility in studies of macrophage function in development. To date, there have been only limited reports of live imaging of macrophages in vertebrate embryos (Herbomel et al., 1999; Colucci-Guyon et al., 2011; Li et al., 2012; Al-Roubaie et al., 2012). We used time-lapse microscopy to visualise the behaviour of embryonic macrophages in response to wounding and stimulation with a microbial-derived particulate antigen. Despite the rapid accumulation of macrophages in regions of programmed cell death and high concentrations of macrophages in the local vicinity of the incisional wound, we did not see any evidence of macrophage recruitment to the wound site. In this respect, the chicken appears to resemble the mouse (Hopkinson-Woolley et al., 1994). One explanation may be the relative lack of cell death at excisional wound sites (Hopkinson-Woolley et al., 1994; Spurlin and Lwigale, 2013), whereas dead cells and macrophages containing dead cells are observed in zebrafish models of wounding (Li et al., 2012). Although embryonic macrophages did not respond to wounding, they were clearly able to recognise and engulf microbes attached to the blood vessel walls (Fig. 4; supplementary material Movie 3). Immediately after engulfment and removal of zymosan particles from the blood vessel wall, several other macrophages were observed patrolling where the zymosan particle had been attached, suggesting some form of chemotactic signalling.

In contrast to imaging of phagocytic cells in quail embryos (Al-Roubaie et al., 2012), we did not observe macrophages integrated into the blood vessel walls in MacReporter chicken embryos. The simplest explanation is that the phagocytic cells integrated into the blood vessel walls in quail are circulating endothelial cells, as suggested previously (Al-Roubaie et al., 2012). In the mouse, yolk sac-derived macrophages do not apparently transit through a monocyte stage, and proliferate extensively as they migrate through the embryo and engulf dying cells (Lichanska and Hume, 2000). Similarly, in the chick, the MacReporter embryo allowed direct observation of dividing macrophages that contain phagocytosed material (supplementary material Movie 3).

Like the *Csf1r*-eGFP (MacGreen) reporter in the mouse (Sasmono et al., 2003), the MacReporter lines in birds allow the visualisation of macrophages *in situ* and, in the adult, they are of special relevance to the delineation of immune-related cells populations. Both the reporter gene and CSF1R were expressed in chicken cells that have been referred to as dendritic cells. Some of these dendritic cells have specific roles in antigen capture and presentation, such as BSDCs and FDCs. The *CSF1R* transgene was also expressed in cells surrounding the splenic ellipsoid, ellipsoid-associated cells (EAC), a phagocytic cell population of haematopoietic origin that functions to remove particulate, immune-complexed and soluble antigen from the blood (Oláh et al., 1984; Igyártó et al., 2007). A significant difference between birds and mammals is the very large number of macrophages in chicken skeletal muscle, detected with the reporter gene. The large resident population of adult skeletal muscle macrophages in MacReporter chickens suggests specific roles for macrophages in muscle development and function.

Birds, like lower vertebrates and monotreme mammals, do not possess lymph nodes and instead have solitary and aggregated lymphoid follicles (Casteleyn et al., 2010). The brightness and specificity of transgene gene expression in the MacReporter chickens enables visualisation of these lymphoid structures in both embryonic and post-hatch chickens. The lymphoid follicles in post-hatch MacReporter chickens are heterogeneous, forming a continuous range of structures ranging from single isolated follicles to aggregates of hundreds of follicles. The MacReporter chicken will provide a model system for the convenient identification and isolation of cells from these lymphoid tissues.

In summary, *CSF1R*-transgene expression in MacReporter chickens allows the chicken mononuclear phagocyte system to be studied with a well-defined marker for the first time. It is a powerful tool for the dynamic visualisation of macrophages in the developing chicken embryo and in post-hatch birds can be used to visualise individual cells of the mononuclear phagocyte system and also the solitary and aggregated lymphoid follicles that represent the majority of secondary lymphoid tissues in the chicken.

## MATERIALS AND METHODS

### Ethics statement

All experiments, animal breeding and care procedures were carried out under license from the UK Home Office and subject to local ethical review.

### Chicken *CSF1R* genomic sequence isolation and plasmid constructs

To define regulatory elements that are sufficient and necessary for gene expression restricted to the mononuclear phagocyte lineage in chickens, a plasmid construct containing 3 kb of the chicken *CSF1R* gene sequence, comprising 2 kb 5′ and 1 kb 3′ of the ATG start codon in the first exon (supplementary material Fig. S1A), was generated by PCR of genomic DNA prepared from whole blood. A modification of the ATG start codon to ATA was also made at this time. The primers 5′-AGTGCAGGCCTGT-GGGGGA-3′ and 5′-GACCAACATCCCCGGGGCCTATGGTG-3′ were designed to amplify the 2 kb 5′ fragment and 5′-ACCCTGCGTGGG-GGCACCATAGGCCC-3′ and 5′-CGCACAGAGGGAAACGCTGC-3′ to generate the 1 kb 3′ fragment using Phusion High-Fidelity DNA Polymerase (Thermo Scientific). Reaction products of the appropriate size were gel purified (PureLink Gel Extraction, Invitrogen) and used in a second round of PCR as template DNA with the primers 5′-AGTGCAGGCCT-GTGGGGGA-3′ and 5′-CGCACAGAGGGAAACGCTGC-3′ to generate a 3 kb product. This 3 kb product was cloned into a pGEM-T Easy vector (Promega) and then subcloned into peGFP-1 (Clontech). This produced two constructs, pMAC.eGFP and pCAM.eGFP, in which the *CSF1R* sequence is in forward or reverse orientation with respect to eGFP (supplementary material Fig. S1B). A further set of constructs were made in which eGFP was replaced with mAPPLE, a modified red fluorescent protein gene (Shaner et al., 2008), to generate pMAC.mAPPLE and pCAM.mAPPLE. As preliminary analysis indicated that pMAC.eGFP did not drive macrophage lineage restricted expression of eGFP (supplementary material Fig. S1C), a further construct was generated in which the FIRE-containing conserved intronic element was subcloned into pMAC.eGFP, downstream of the promoter element (supplementary material Fig. S1B). This FIRE-containing conserved intronic element was generated by PCR of genomic DNA using the primers 5′-AGAAAGATAAAAGCATTGCACA-3′and 5′-CCCCATT-TTGCCACATCAGCGAG-3′ to produce an 820 bp product, using Thermo Scientific Phusion High-Fidelity DNA Polymerase. This was cloned into pGEM-T Easy vector (Promega) and subcloned into pMAC.eGFP, positioned 3′ to the 3 kb insert and 5′ to eGFP to produce the construct pMAC.FIRE.eGFP (supplementary material Fig. S1B). This produced two pMAC.FIRE constructs, pMAC.FIRE.eGFP and pMAC.FIRE.mAPPLE. A further modification was made at this point with a splice acceptor sequence (5′-GGGCCCGATTTTTTTTCATCCTCATTTTTCTCTTTCCT-TTGCAGGCTCCACCGGT-3′) being sub-cloned into the *Apa*I-*Age*I site immediately 5′ of the eGFP/mAPPLE ATG start codon to produce pMAC.FIRE.SA.eGFP and pMAC.FIRE.SA.mAPPLE.

### Cell lines and transfection experiments

HD11 is a chicken macrophage cell line derived from bone marrow cells transformed with an avian myelocytomatosis virus (Beug et al., 1979). DF-1 is a spontaneously immortalised chicken embryo fibroblast cell line (Himly et al., 1998). Both cell lines were cultured in RPMI 1640 medium containing 20 mM L-glutamine (Life Technologies), 10% newborn calf serum, 2.5% chicken serum supplemented with penicillin-streptomycin at 41°C in 5% CO_2_. Cells (5×10^6^) were transfected with 10 μg of each reporter construct (supplementary material Fig. S1B) by electroporation at 280 V and a capacitance of 960 μF, using a Bio-Rad Gene Pulser. For stable transfections, cells were pelleted and washed with medium to remove DNA. After re-suspension, the cells were split into three independent pools and cultured for 48 h without selection, washed and cultured with for selection using 200 μg/ml of G418 (geneticin; Gibco BRL) for 17-32 days.

### Construction of lentiviral vectors

The pLenti6/R4R2/V5-DEST vector (Invitrogen) was modified by removal of the blasticidin-containing *Kpn*I-*Pml*I-containing fragment and the addition of a woodchuck hepatitis virus post-transcriptional regulatory element optimized for safety (oPRE). The *CSF1R* reporter gene was isolated from pMAC.FIRE.mAPPLE using *Xba*I and *Xho*I, blunt-ended using Klenow DNA polymerase and subcloned into the modified pLenti6/R4R2/V5-DEST to produce pLenti.MAC.FIRE.mAPPLE. In order to add a splice acceptor site, pMAC.FIRE.SA.mAPPLE was cut with *Mfe*I, blunt-ended using Klenow DNA polymerase and then digested with *Eco*RV. The fragment containing partial *CSF1R*-splice acceptor-mAPPLE sequence was gel purified. pLenti.MAC.FIRE.mAPPLE was digested with *Eco*RV to release a fragment containing the *CSF1R*/mAPPLE sequence. The gel-purified pMAC.FIRE.SA.mAPPLE splice acceptor sequence fragment was then subcloned into *Eco*RV-digested pLenti.MAC.FIRE.mAPPLE to produce pLenti.MAC.FIRE.SA.mAPPLE. An identical strategy was used to produce pLenti.MAC.FIRE.SA.eGFP (supplementary material Fig. S1D).

### Preparation of viral stocks

Vector stocks were generated by FuGENE6 (Roche) transfection of HEK 293T cells plated on 10 cm dishes with 3 μg pLenti.MAC.FIRE.SA.mAPPLE/pLenti.MAC.FIRE.SA.eGFP, 6 μg HIV gag/pol plasmid (psPAX2, Addgene) and 1.6 μg of VSV-G (pLP/VSV-G, Invitrogen) plasmid per plate. At 36-48 h after transfection supernatants were filtered (0.22 μm). Concentrated vector preparations were made by initial low-speed centrifugation at 6000 ***g*** for 16 h at 4°C followed by ultracentrifugation at 50,500 ***g*** for 90 min at 4°C. The viral particle pellet was resuspended in 60-80 μl of medium (McGrew et al., 2004).

### Production and analysis of transgenic birds

Approximately 1-2 μl of viral suspension was microinjected into the subgerminal cavity beneath the blastodermal embryo of newly laid eggs. Embryos were incubated to hatch using phases II and III of the surrogate shell *ex vivo* culture system (Perry, 1988). DNA was extracted from the chorioallantoic membrane (CAM) of embryos that died in culture at 12 days of development or more, using the Puregene genomic DNA purification kit (Flowgen). Genomic DNA samples were obtained from CAM of G_0_ chicks at hatch, blood samples from older birds and semen from mature cockerels (supplementary material Fig. S1E,F). PCR analysis was carried out on 50 ng DNA samples for the presence of proviral sequence. To estimate copy number, control PCR reactions were carried out in parallel on 50 ng aliquots of chicken genomic DNA with vector plasmid DNA added in quantities equivalent to that of a single-copy gene (1×), a tenfold dilution (0.1×) and a 100-fold dilution (0.01×) as described previously (Sherman et al., 1998). Primers used were as follows: HIV1 5′-GAGAGAGATGGGTGCGAGAG-3′ and HIV2 5′-GCTGTGCGGTGGTCTTACTT-3′. Deletion of FIRE in transgenic birds was assessed by PCR using primers P1 5′-ACAACCAG-AAGGGGAAGGTGG-3′ and P2 5′-GTCGGGGATGTCGGCTGGGT-3′ (supplementary material Fig. S1D) using conditions outlined above. The number of proviral insertions and size of inserts in individual G_1_ birds was analysed by Southern blot transfer. Genomic DNA extracted from whole blood was digested with *Xho*I and *Cla*I (supplementary material Fig. S1D). The digested DNA was resolved on a 0.6% (w/v) agarose gel and then transferred to a nylon membrane (HybondN). Membranes were hybridized with ^32^P-labelled probes for the reporter gene mAPPLE or eGFP at 65°C. Hybridization was detected by autoradiography (supplementary material Fig. S2A). All experiments, animal breeding and care procedures were carried out under license from the UK Home Office and subject to local ethical review.

### Embryonic staging

Embryos were assigned a Hamburger–Hamilton (HH) stage based on previously defined criteria (Hamburger and Hamilton, 1951).

### *CSF1R*-transgene expression analysis

#### Confocal analysis

Embryonic and adult tissues were isolated, fixed for 1 h to overnight in 4% paraformaldehyde in phosphate-buffered saline (PBS), washed in PBS and perfused overnight in 15% sucrose in PBS. Selected samples were then were cryo-embedded in Tissue-Tek OCT compound (Sakura Finetechnical) and sectioned at 10 μm onto Superfrost Plus (Menzel-Gläser) slides. Sections were blocked for 1 h in 10% skim milk powder, 10% normal horse serum, 0.1% Triton X-100 in PBS (MST-PBS). Primary antibodies were added: anti-CSF1R (Garcia-Morales et al., 2013); anti-MHC II [clone 2G11 (Kaufman et al., 1990)]; anti-chicken CD41/61 (clone 11C3, AbD Serotec); CD45 (clone LT40, SouthernBiotech); anti-Bu-1 (clone L22, AbD Serotec); anti-chicken macrophage subset marker (clone CVI-ChNL-74.2, Prionics); anti-chicken macrophage/monoctyes (clone KUL01, AbD Serotec); and anti-chicken TCR alpha/beta (clone TCR2, AbD Serotec) all diluted by 1/50-1/500 in MST-PBS and sections incubated at 4°C overnight. Sections were then washed for 30 min in PBS and re-incubated with secondary antibodies diluted 1/300 in MST-PBS for 1 h (goat anti-rabbit IgG Alexa Fluor 488, donkey anti-mouse IgG Alexa Fluor 543; Life Technologies), then washed for 30 min in PBS and mounted in Hydromount (National Diagnostics). In some cases the sections were counterstained with the addition 1 μg/ml 4′,6′-diamidino-2-phenylindole (Sigma) in the final incubation step. For visualising epidermal mononuclear phagocyte populations, areas of featherless skin from the neck region were cut (1.0×1.0 cm^2^) and incubated in RPMI medium (Sigma) containing 2 mg/ml dispase (grade II, Roche) for 1 h at 37°C. After incubation, the epidermis was lifted from the dermis, using sterile forceps and washed in RMPI media. The epidermal sheet was mounted on a plastic Petri dish and overlaid with sterile PBS. Cells were imaged using an inverted confocal microscope (Nikon eC1, Nikon Instruments). Images were captured using Nikon EZ-C1 Software v3.40.

#### Flow cytometry

Flow cytometry was performed to characterise the *CSF1R*-transgene-expressing cells using mouse monoclonal antibodies to chicken CD3 (clone CT3, AbD Serotec), Bu-1 (clone AV20, AbD Serotec), CD45 (clone AV53, Institute for Animal Health, UK), KUL01 (SBA, SouthernBiotech), CSF1R (Garcia-Morales et al., 2013) and MHC II [clone 2G11 (Kaufman et al., 1990)]. Cells were stained for 30 min at 4°C, washed with PBA (PBS, 0.5% BSA and 0.05% sodium azide) followed by incubation with a goat-anti-mouse-IgG1-alexa 647 (Invitrogen) for 30 min at 4°C. Cells were washed with PBA and analysed using a FACS Calibur flowcytometer (BD Biosciences). At least 100,000 events were acquired in the lymphocyte gate and data were analysed using the software program FlowJo (Threestar).

#### Whole-mount fluorescence imaging

For images of embryonic tissues, embryos were either imaged *in ovo* or removed from the egg and placed in PBS and imaged. In the former case, 10% Indian ink (Winsor & Newton) solution in PBS was injected underneath the embryo into the yolk sac to block autofluorescence. Lymphoid tissues in embryonic and post-hatch birds were imaged by dissecting the relevant organ, which was placed in a Petri dish for imaging.

### Chicken embryo and organ culture and time-lapse imaging

Embryos were cultured using a modified EC culture (Chapman et al., 2001). HH16/17 stage embryos were removed from eggs using sterilised Whatman 3MM CHR filter paper rings, cut into rings to fit the internal diameter of six-well plates (Costar, Corning), washed in HBSS and placed on an albumen/agar plate ventral side upwards. For limb bud culture, hind limb buds were dissected from HH31 stage embryos and placed in a six-well albumen/agar plate. After wounding (see below) limb buds were embedded in a thin layer of amniotic fluid/agar. Amniotic fluid was removed from embryos prior to dissection of limb buds using a sterile needle and syringe. Plates were left in a fully humidified 38°C incubator for 1 h to allow for settling of the embryo. Embryos were scanned every 5 min for the period of culture using a Nikon TiE (Perfect Focus System) microscope with NIS-Elements 4.0 equipped with an incubation chamber at 38°C, 100% humidity. Images were compiled and merged using public domain software ImageJ v.1.41o (NIH).

### Embryo wounding

Cuts were made in the eye primordium and of limb buds of embryos using an ultrafine tungsten dissecting needle (Harvard Apparatus UK) with a 1 µm tip diameter (Brock et al., 1996). Crush wounds were produced by pinching the distal limb bud of HH31 stage embryos using jewellers forceps. For eye primordium wounding, the tip of the needle was inserted into the lens vesicle and used to produce a cut extending through to the outer edge of the optic cup of HH16 stage embryos *in ovo*. Embryos were either incubated *in ovo* for 24 h or removed from eggs for live imaging (see above). For organ culture limb bud wounding, after dissected limb buds had been placed on albumin/agar plates the needle was used to produce a cut in the tip of the middle digit. Limb buds were then cultured as described above. For *in ovo* limb bud wounding, the tip of HH31 stage embryos was either cut with a tungsten needle or crushed using jewellers forceps. Embryos were then incubated for a further 24 h before imaging.

### Bioinformatics analysis

The *CSF1R* sequence was analysed using the software mVista alignment (http://gsd.lbl.gov/vista/) and MacVector (http://macvector.com/). Nucleotide sequences were identified using the databases at the National Center for Biotechnology Information (Bethesda, MD, USA), the genome resources from the University of Santa Cruz (Santa Cruz, CA, USA) and Ensembl (www.ncbi.nlm.nih.gov/index.html, http://genome.ucsc.edu and www.ensembl.org/index.html), and the Beijing Genome Institute (BGI) Bird Phylogenomic Project (http://phybirds.genomics.org.cn/).

### *CSF1R* orthologues

*CSF1R* orthologues were as follows: human (*Homo sapiens*), GRCh37:5:149432254:149493535:1; mouse (*Mus musculus*), GRCm38:18:61104972:61132749:1; platypus (*Ornithorhynchus anatinus*), OANA5:X1:29260121:29291367:1; Chinese softshell turtle (*Pelodiscus sinensis*), PelSin_1.0:JH224652.1:1894358:1933352:-1; alligator (*Alligator mississippiensis*), GenBank AKHW01092331.1, scaffold-11218_4; chicken (*Gallus gallus*) Galgal4:13:12593807:12612065:1; turkey (*Meleagris gallopavo*), NW_003436014.1, chromosome 15 genomic scaffold, Turkey_2.01; zebra finch (*Taeniopygia guttata*), Chr13: 6,954,381–6,972,446; July 2008 assembly; GQ249407; Adélie penguin (*Pygoscelis adeliae*): Scaffold34:2101303:2117750; budgerigar (*Melopsittacus undulatus*), Adam_Phillippy_v6_sli_scf900160277035:2037315:2055959; ostrich (*Struthio camelus*), scaffold80:1343069:1358427; rifleman (*Acanthisitta chloris*): scaffold10495:21554:35197; duck (*Anas platyrhynchos*), scaffold111:241:9980 (sequences available from BGI Bird Phylogenomic Project).

## Supplementary Material

Supplementary Material
