# Supplementary material for: Visualisation of chicken macrophages using transgenic reporter genes: insights into the development of the avian macrophage lineage
Source: Development. 2014 Aug;141(16):3255–65. doi: 10.1242/dev.105593 (PMC4197536; doi:10.1242/dev.105593)
Supplement: Supplementary Material [file supp_141_16_3255__index.html]

Visualisation of chicken macrophages using transgenic reporter genes: insights into the development of the avian macrophage lineage — Supplementary Material 

# Visualisation of chicken macrophages using transgenic reporter genes: insights into the development of the avian macrophage lineage

## DEV105593 Supplementary Material

**Files in this Data Supplement:**

- **Supplementary Material**
