# Supplementary material for: Visualisation of chicken macrophages using transgenic reporter genes: insights into the development of the avian macrophage lineage
Source: Development. 2014 Aug;141(16):3255–65. doi: 10.1242/dev.105593 (PMC4197536; doi:10.1242/dev.105593)
Supplement: Supplementary Material [file supp_141.16.3255_DEV105593.pdf]

Figure S1. **Stable transfection analysis of the activity of chicken *CSF1R* regulatory elements, using eGFP reporter gene constructs.** Cells were transfected with 10 µg of reporter plasmid by electroporation stably transfected cells were isolated by geneticin selection (see Supplementary Experimental Procedures). (A) Schematic representation of the chicken *CSF1R* genomic organisation upstream of exon 2, showing the position of the ATG start codon in the first exon, and (B) the plasmid constructs used in this study. (C) eGFP expression in HD11 chicken macrophage-like and DF-1 chicken fibroblast cell lines after stable transfection with: i) pEGFP-1, ii) pMAC.eGFP, iii) pCAM.eGFP or iv) pMAC.FIRE.eGFP plasmid vectors. (D) Schematic representation of the chicken *CSF1R* genomic organisation upstream of exon2, and of the HIV vector used in this study. The restriction enzyme sites (ClaI and XhoI) and PCR primer (P1 and P2) locations used in subsequent analysis (Fig. S2) are shown. LTR = long terminal repeat. (E) Number of G0 cockerels produced (MAA = mApple transgene; MAG = eGFP transgene). (F) Analysis of germline transmission from G0 cockerels by PCR analysis and number of PCR+ G1 progeny expressing the fluorescent protein reporter.

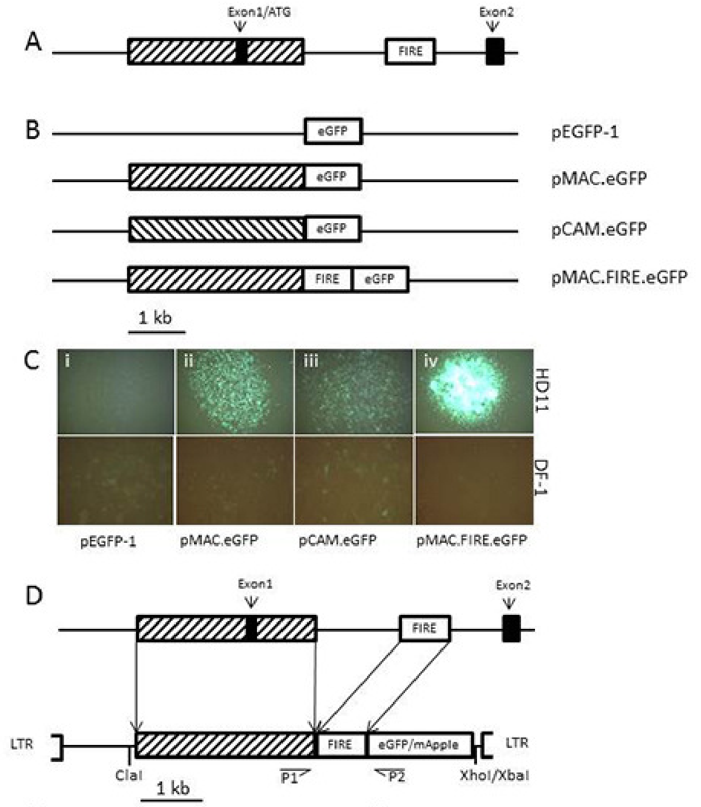

E

Expert. No.	No. eggs injected	Hatched chicks (G_0)	M/F
MAA1	12	2	2/0
MAA2	15	2	2/0
MAA3	27	0	-
MAA4	18	3	2/1
MAG1	23	4	1/3
MAG2	21	6	4/2

F

Cockerel ID	Genome equivalents semen	Germline transmission Total screened /transgenics	No. chicks with fluorescent cells
MAA 2.16	100%	276/104 (38%)	11 (4%)
MAA 1.7	100%	70/15 (21%)	0 (0%)
MAA 1.8	100%	66/33 (50)%	1 (1.5%)
MAG 1-9	100%	956/197 (21%)	3 (0.3%)
MAG 2-8	100%	61/13 (21%)	0 (0%)

Figure S2. **Expression of fluorescent protein reporter in MPs requires integration of a full length *CSF1R*-reporter construct containing chicken FIRE**. (A) Southern blot analysis of G1 progeny from G0 founder MAA2-16. DNA isolated from the blood of four G1 birds (lanes 1-4) were digested with restriction enzymes XhoI and ClaI isolate the full-length insert and probed with sequence mApple coding sequence. Expected size of full length insert (5.5 kb) was observed in G1 progeny MAA2-16:13 and MAA2-16:22 (lane 1 and 3), whereas a partially-deleted insert was found in G1 progeny MAA2-16:20 and MAA2-16:33. (B) PCR analysis of G2 progeny (lanes 1-12) from G1 cockerel MAA1-8:23 for the presence of full length (2.5 kb) or partially-deleted (0.5 kb) inserts using primers P1 and P2. (C) Flow cytometric analysis of reporter transgene expression in KUL01+/CSF1R+ blood monocytes in a non-transgenic chicken, G1 cockerel MAA1-8:23 and representative G2 progeny from (B) that have either a full length or a partially-deleted insert.

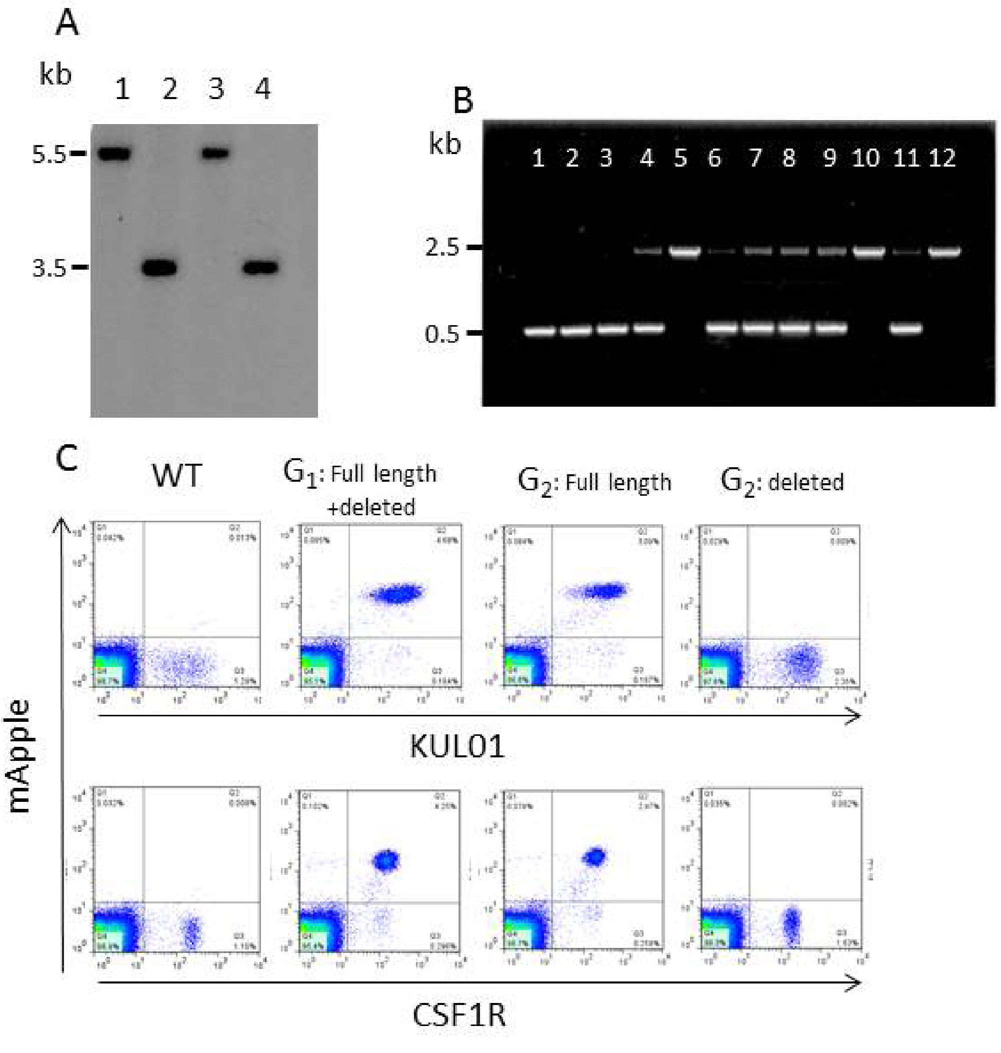

Figure S3. Flow cytometric analysis of transgene expression in blood cell populations Transgene expression in R1 (lymphocytes/monocytes) and R2 (heterophils) cell populations from a representative G1 transgenic bird (MacRed, MAA2-16:22) in comparison to a non-transgenic bird. (A) Analysis of transgene expression in R1 (lymphocytes/monocytes); (B) Analysis of transgene expression in R2 (heterophils). High transgene expression is restricted to the KUL01+/MHCII+/CD11+/CSF1R+ monocyte population. Low level expression of *CSF1R*-mApple and CSF1R was also observed in the heterophil population. Analyses were carried out on samples from at least 3 birds with different, single integrated copies of the transgene.

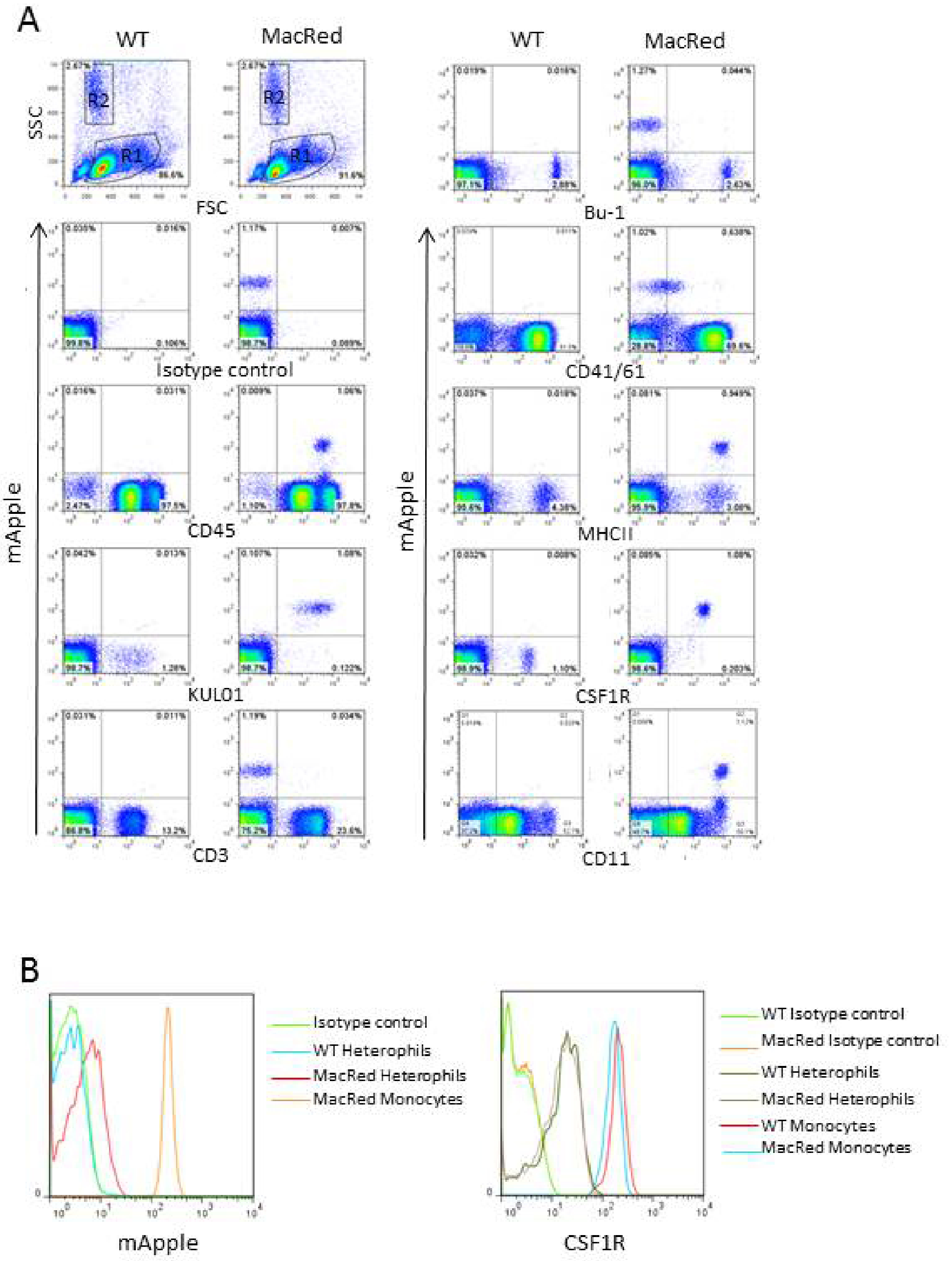

Figure S4. High level expression of CSF1R on BSDC and FDC MP subsets. Immunofluorescence staining of CSF1R of week 10 chicken bursa of Fabricius (A) and caecal tonsils (B). CSF1R staining is characteristic of BSDC and FDC populations. Scale bars: 200 µm.

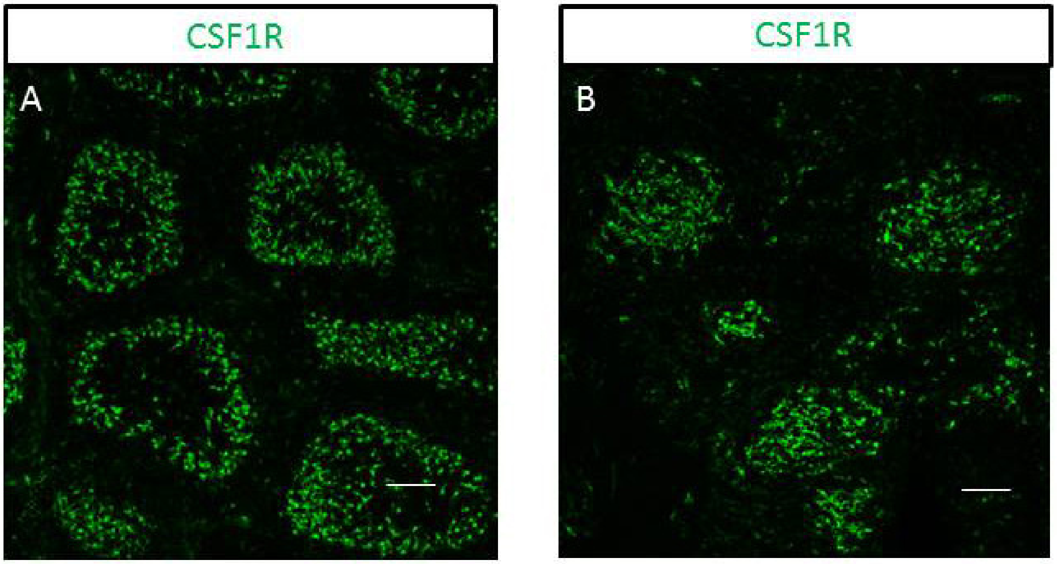

Movie 1. Embryonic macrophages do not accumulate at wounds in chicken embryos. Time-lapse imaging of the a MacGreen HH16 chicken embryo taken for five hours after an incisional wound was made in the eye primordium (arrow). While a few macrophage (green) are located in the general area of the wound, there is no further accumulate macrophages during the period of filming. Asterisk (*) marks large accumulation of macrophages in the supraorbital region. Scale bar: 100 µm.

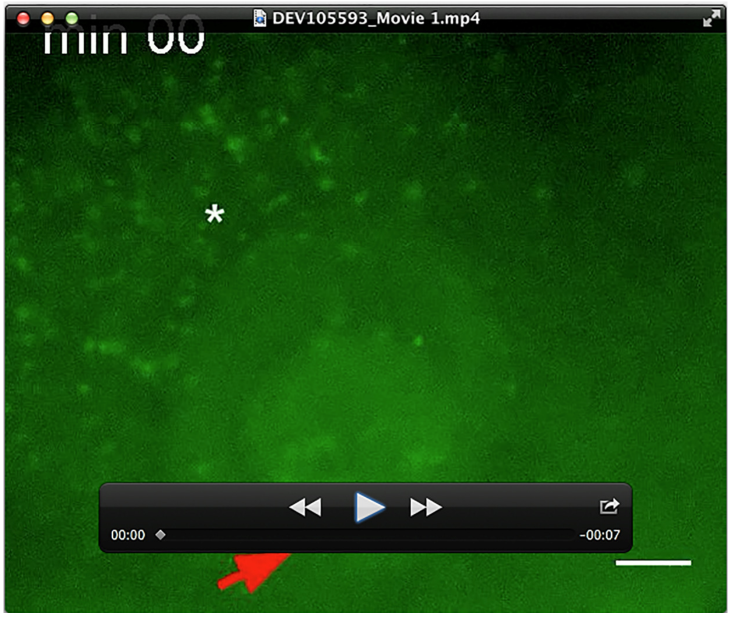

Movie 2. Embryonic macrophages associated with the embryonic vasculature are not integrated into blood vessels. Time-lapse imaging of region of the vitelline vasculature in a MacGreen HH17 embryo. Imaged for seven hours at five minute intervals. Macrophages associated with blood vessels are highly dynamic and move along blood vessel in both clusters of cells (red arrow) and single cells (blue arrow). Scale bar: 200 µm.

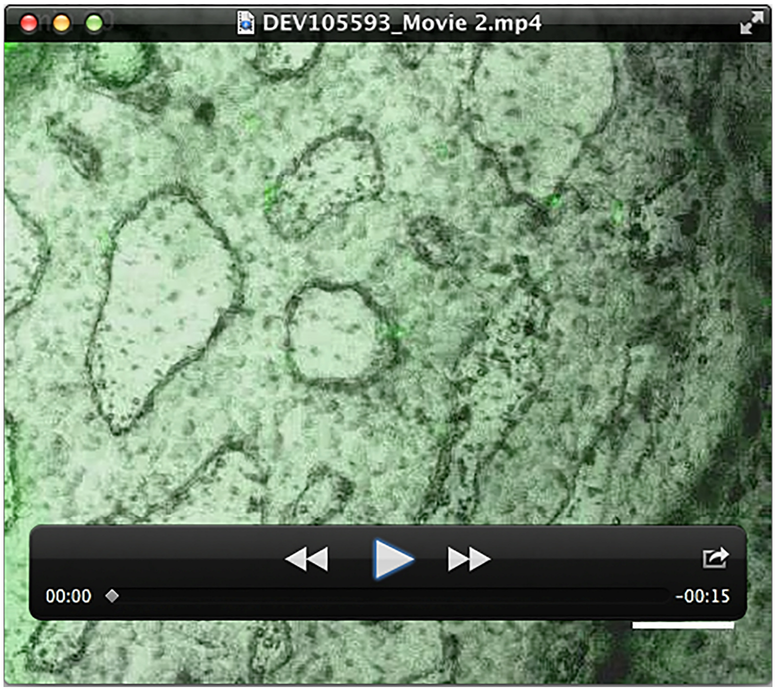

Movie 3. Macrophages associated with the embryonic vasculature are highly motile, phagocytic and undergo local division. A: Time-lapse imaging of region above the vitelline artery near embryo proper. Imaged for five hours at five minute intervals. The aorta of *CSF1R*-eGFP embryos was injected with Texas Red-labelled zymosan one hour prior to the beginning of imaging. Most Zymosan particles adhere to the blood vessel walls (yellow arrow). $eGFP^+$ macrophages are highly motile. Between 100 and 125 min a zymosan particle becomes associated with a macrophage (yellow arrow), this macrophage re-enters the circulation, removing the zymosan particle by 150 min. At 0 min a zymosan particle is contained within a macrophage (white arrow), from 0-75 min this macrophage is both motile and exhibits changes in morphology. At 100 min this macrophage (white arrow) no longer exhibits movement and does not extent any cellular processes. A similar macrophage without a phagocytised zymosan particle (blue arrow) exhibits identical behaviour. At 100-150 min both undergo division (white and blue arrows) and daughter cells resume active patrolling the vasculature. Scale bar: 50 µm.

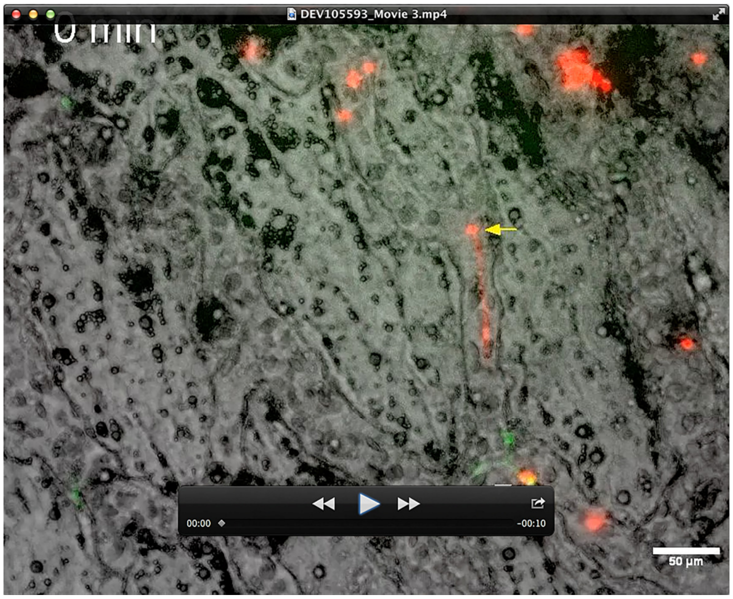
DEV105593_Movie 3.mp4
0 min
00:00
-00:10
50 µm
